# Single‐Use Versus Multiple‐Use Endotracheal Suction Catheters in Mechanically Ventilated Patients: A Feasibility Randomised Controlled Trial

**DOI:** 10.1111/nicc.70237

**Published:** 2026-01-07

**Authors:** Mohamed H. Eid, Kevin Hambridge, Patricia Schofield, Jos M. Latour

**Affiliations:** ^1^ School of Nursing and Midwifery, Faculty of Health University of Plymouth Plymouth UK; ^2^ Critical Care and Emergency Nursing Department, Faculty of Nursing Mansoura University Mansoura Egypt; ^3^ The Curtin School of Nursing Curtin University Perth Western Australia Australia; ^4^ Department of Nursing Zhongshan Hospital, Fudan University Shanghai China

**Keywords:** catheters, chlorhexidine, endotracheal, equipment reuse, intensive care units, suction

## Abstract

**Background:**

In resource‐limited settings, single‐use open endotracheal suction catheters are commonly used multiple times. The current evidence of this practice on ventilator‐associated pneumonia (VAP) among mechanically ventilated patients remains unclear.

**Aim:**

The aim of this study was to test the feasibility of single‐use and multiple‐use endotracheal suction catheters flushed with chlorhexidine versus standard care (multiple‐use endotracheal suction catheters flushed with normal saline) to reduce VAP in resource‐limited intensive care units (ICUs) and evaluate study methods for designing a future definitive randomised controlled trial (RCT).

**Study Design:**

A three‐armed feasibility RCT was conducted in three ICUs at a university hospital in Egypt.

**Results:**

Sixty mechanically ventilated patients were randomized into three groups: Intervention I group, single‐use catheters, Intervention II group, multiple‐use catheters flushed with chlorhexidine, and Control group (standard care) multiple‐use catheters flushed with normal saline. Data on feasibility parameters, intervention adherence, and patient outcomes were collected. Recruitment and retention rates were high across all groups, with 96.7% (*n* = 58) of participants completing the 3‐day follow‐up and 93.3% (*n* = 56) completing the 6‐day follow‐up. Adherence to intervention protocols was excellent, with 100% of participants receiving the designed interventions. No adverse events were reported. Chlorhexidine flushing and single‐use catheters were practical and successfully implemented. Challenges of budget constraints, supply shortages and the need for staff training were observed. The incidence of VAP was 31.6% in the Intervention I group, 26.3% in the Intervention II group, and 40% in the standard care group. The relative risk of developing VAP compared with the control group was 0.79 (95% CI: 0.51–1.23) for Intervention I and 0.66 (95% CI: 0.42–1.03) for Intervention II.

**Conclusions:**

A full‐scale RCT comparing single‐use and multiple‐use catheters with chlorhexidine flushing is feasible in resource‐limited ICUs. Further investigation into the effectiveness of interventions on VAP prevention is needed in future definitive RCTs.

**Relevance to Clinical Practice:**

This study highlights the practical implementation of endotracheal suctioning strategies, such as chlorhexidine flushing and single‐use suction catheters, to potentially reduce VAP in resource‐limited ICUs. These findings can inform clinical decision‐making and infection control.

**Trial Registration:** ClinicalTrials.gov, identifier NCT06207513

**Impact Statements**

- What is known about the topic
  ○ In resource‐limited settings, single‐use endotracheal suction catheters are commonly used multiple times for suctioning.
  ○ Evidence on the impact of this practice on ventilator‐associated pneumonia (VAP) in mechanically ventilated patients is limited.
  ○ Nurses should adhere to fundamental sterile techniques before, during and after endotracheal suction procedures.
- What this paper adds
  ○ This study shows the feasibility of conducting a full‐scale randomised controlled trial (RCT) comparing single‐use and multiple‐use suction catheters with chlorhexidine flushing in resource‐limited ICUs.
  ○ It provides data on VAP incidence and ICU length of stay, informing the design of a future multicentre trial.
  ○ Single‐use and multiple‐use endotracheal suction catheters with chlorhexidine flushing in resource‐limited ICUs might reduce VAP, but these practices need to be confirmed with robust new evidence.

## 1 Introduction

The debate over single‐use versus reusable equipment in intensive care units (ICUs) has gained global attention, particularly due to concerns about environmental impact [1]. In resource‐limited settings, reusable equipment is more commonly used, especially in low‐ and middle‐income countries where healthcare resources are constrained. One example of such equipment is open endotracheal suctioning catheters, which are used multiple times for endotracheal suctioning in ICUs with limited resources [2, 3]. However, the current evidence regarding this practice and its effects on the outcomes of mechanically ventilated patients remains unclear.

## 2 Background

Mechanically ventilated patients are prone to increased secretion build‐up due to sedation and the use of ventilator mechanisms, which prevent the body's natural ability to clear secretions [4]. As a result, endotracheal suctioning has become a standard practice in respiratory care to manage these secretions in ventilated patients [5, 6]. This procedure not only helps remove pulmonary secretions but also enhances oxygenation and ventilation while reducing the risk of complications, such as pneumonia, atelectasis and endotracheal tube obstruction caused by secretion retention [7].

A suction catheter is essential for clearing tracheal secretions and can be integrated into either an open or closed endotracheal suctioning system [8]. In the open endotracheal suctioning system, the ventilation circuit is temporarily disconnected to insert a disposable suction catheter, whereas the closed system maintains the catheter within a sterile, enclosed sheath attached to the endotracheal tube, eliminating the need for disconnection [9]. Recent research shows no significant difference in the incidence of ventilator‐associated pneumonia (VAP) or mortality rates between the two systems [8, 9, 10, 11]. Furthermore, a randomised trial found that replacing closed endotracheal suction catheters every 3 days versus every 7 days did not impact VAP rates [12].

Endotracheal suctioning should not be performed on a routine schedule but rather based on the patient's clinical needs. Indications for suctioning include visible airway secretions, coughing and the presence of coarse crackles heard over the trachea during auscultation [9]. As a sterile procedure, endotracheal suctioning must be carried out following specific guidelines to minimise the risk of lung contamination. Therefore, nurses should adhere to fundamental sterile techniques before, during and after the procedure [13]. These principles are outlined in the latest clinical practice guidelines on endotracheal suctioning published by the American Association of Respiratory Care [14].

Chlorhexidine is a widely recognised, cost‐effective disinfectant known for its ability to eliminate most pathogenic microorganisms, helping to reduce the transmission of hospital‐acquired infections in ICUs [15]. It is endorsed by leading health organisations, such as the American Thoracic Society (ATS), the Centres for Disease Control and Prevention (CDC), and the Infectious Diseases Society of America (IDSA). One of its key features is its prolonged antibacterial effect, which can last up to 48 h after application. This sustained activity prevents the growth of microorganisms that come into contact with chlorhexidine during this period [16]. This highlights the importance of exploring how chlorhexidine can be effectively incorporated into practices like endotracheal suctioning, where evidence and guidelines remain limited.

There is no compelling evidence of the frequency of changing endotracheal suction catheters. Nurses in resource‐limited countries follow their hospital policy regarding the changing frequency of open endotracheal suction catheters due to a lack of robust evidence [17]. One study suggested that flushing suction circuits with chlorhexidine while reusing single‐use catheters might reduce the risk of respiratory infections in mechanically ventilated patients [2]. Therefore, we initiated a feasibility study to contribute to this gap in the literature.

### 2.1 Aim and Objectives

The aim of this feasibility randomised controlled trial (RCT) was to determine the feasibility of investigating the effect of single‐use or multiple‐use of open endotracheal suction catheters flushed with chlorhexidine compared to standard care of multiple‐use open endotracheal suction catheters flushed with normal saline on VAP incidence in mechanically ventilated patients to determine methods for the design of a conclusive RCT.

The objectives of this study were to: (1) assess the feasibility and acceptability of the trial procedures; (2) select the most appropriate primary and secondary outcome measures to inform sample size calculation of the future RCT; (3) identify confounding factors that might affect the future trial; and (4) assess patients' recruitment technique, follow‐up rate and any unexpected side effects of the intervention.

## 3 Methods

This study adopted a three‐armed feasibility RCT design. Recruitment took place between April and December 2024. The study protocol was published prospectively [18]. This paper will present the method and quantitative findings of the feasibility RCT, whereas the findings of the embedded qualitative study will be reported in a separate paper. The CONSORT 2010 statement: extension for randomised pilot and feasibility trials [19] was used to report this study.

### 3.1 Ethical Considerations

Ethics approval was obtained from two ethics committees: the Faculty of Health Research Ethics and Integrity Committee at the University of Plymouth, United Kingdom (Reference: 4333, Dated 14, December 2023) and the Research Ethics Committee at the Faculty of Nursing, Mansoura University, Egypt (Reference: 411, Dated 27 November 2023). The study protocol was prospectively registered at ClinicalTrials.gov with the number: NCT06207513.

### 3.2 Setting

This study was conducted in three ICUs within a university hospital in Egypt. The ICUs included surgical, neurological and trauma units, each consisting of 10 beds and providing care to mechanically ventilated patients. Collectively, the three ICUs admit approximately 650 patients per year. The nurse‐to‐patient ratio in these units is maintained at one nurse for every two patients. Standard VAP prevention measures in these ICUs include maintaining the head of the bed at an angle between 30 and 45 degrees, conducting daily sedation vacations and weaning assessments, and implementing prophylaxis for peptic ulcer disease, which adhere to the ventilator bundle checklist established by the Institute for Healthcare Improvement (IHI, 2012) [20].

### 3.3 Participants

The study included adult patients aged 18 years and older who were newly admitted to the ICU, intubated with an endotracheal tube and expected to require mechanical ventilation for a minimum of 48 h. The exclusion criteria were as follows: (1) patients who have already received standard ICU care involving multiple endotracheal suctioning procedures; (2) patients with contraindications to suctioning, including increased intracranial pressure, severe haemoptysis or cerebrospinal fluid leaks; (3) patients who have been intubated previously during their current hospital stay; (4) patients anticipated to need mechanical ventilation for less than 72 h; (5) patients diagnosed with pneumonia at ICU admission or those with a Modified Clinical Pulmonary Infection Score (MCPIS) of 5 or above; (6) patients with conditions, such as atelectasis, acute respiratory distress syndrome (ARDS) or pulmonary oedema because of their disease pathophysiology; (7) patients with a known allergy to chlorhexidine and (8) patients whose next of kin have not provided deferred consent within 48 h of ICU admission.

### 3.4 Sample Size and Randomisation

As the study design is a feasibility RCT, no formal power calculation was done. According to guidelines for feasibility studies, the recommended sample size is usually between 20 and 70 participants [21].

A sealed envelope system was used as the randomisation technique to allocate participants, and this method was assessed in the feasibility RCT to inform the randomisation approach for a future RCT. In this method, opaque, pre‐prepared envelopes containing group assignments generated from a random sequence were thoroughly shuffled and securely stored. The clinical nurse lead opened an envelope for each participant at enrolment to reveal the allocation, ensuring allocation concealment and allowing assessment of the method's feasibility [22]. In this feasibility RCT, this approach was used to test whether this simple, practical method would effectively maintain random allocation and concealment for a future, larger trial.

Blinding was not feasible in this trial due to differences in the bottle shape and slight colour variations between chlorhexidine and normal saline solutions. Patient screening, consent and recruitment were assigned to the clinical nurse lead, whereas the principal investigator (PI; MHE) was responsible for collecting the outcome measures. Critical care nurses performed the interventions, ensuring the appropriate delivery of care for each study group.

### 3.5 Recruitment

Recruitment was carried out by the clinical nurse lead or the PI. An initial assessment was conducted for all newly admitted mechanically ventilated patients on their first day in the ICU using the MCPIS to ensure they were free from pneumonia and met the inclusion criteria. We aimed to complete allocation before the first endotracheal suctioning procedure to prevent contamination and crossover, as each study group followed a distinct suctioning technique.

Deferred consent was obtained from the patients' families (next of kin), who were provided with information regarding the study's purpose, procedures, benefits and risks. Initial verbal consent was accepted, and the family was given a hard copy of participant information sheets and consent forms. Written consent was collected within 24 h of distributing the patient information sheet. The voluntary nature of participation and the right to withdraw at any time without obligation were clearly communicated. Confidentiality and anonymity of participants' personal information were maintained throughout the study by utilising unique study identification codes.

### 3.6 Nurse Training

The PI offered training on how to conduct study interventions to the ICU nurses at the study sites prior to implementing the intervention. Once the PI confirmed the nurses' competency, they could commence the intervention delivery. Additional information regarding this training is available in Data S1.

### 3.7 Interventions and Control

Study participants were randomly assigned using a sealed envelope system randomisation technique to one of the following three groups:

#### 3.7.1 Intervention I Group (Single‐Use Endotracheal Suction Catheter)

The ICU nurses performed endotracheal suctioning for this group using a single‐use open endotracheal suction catheter. Each catheter was used for only one suctioning attempt and discarded immediately after removal from the patient. If additional suctioning was needed, a new catheter was used and subsequently discarded. To ensure continuity of care for this group, a red label was applied to their folders for clear identification.

#### 3.7.2 Intervention II Group (Multiple‐Use Endotracheal Suction Catheter Flushed With Chlorhexidine)

For this group, ICU nurses performed endotracheal suctioning using an open endotracheal suction catheter that was reused multiple times during a 12‐h nursing shift. A separate catheter was designated for the day shift and another for the night shift. The suctioning circuit was flushed with 40 mLs of 0.2% chlorhexidine gluconate after every suctioning procedure. Before reinserting the catheter, nurses conducted 5 s of ‘dry suctioning’ to ensure the removal of any residual chlorhexidine droplets, preventing accidental instillation into the patient's lungs. To ensure continuity of care for this group, a yellow label was applied to their folders for clear identification.

#### 3.7.3 Control Group (Standard Care)

Following the hospital policy, ICU nurses performed their standard suctioning procedure for this group, using an open endotracheal suction catheter that was reused multiple times within a 12‐h nursing shift, with separate catheters assigned for the day and night shifts. The suctioning circuit was flushed using 40 mL of normal saline after every suctioning procedure. To ensure continuity of care for this group, a green label was applied to their folders for clear identification.

### 3.8 Data Collection

Data collection in this study involved three distinct tools (Data S2).

Quantitative data were obtained from participant patients using two specific tools, as summarised in Box 1.

**BOX 1:** Summary of quantitative data collection tools.

Tool Ι: Mechanically Ventilated Patients Assessment Tool
This tool was designed by the principal investigator (PI) following a comprehensive review of relevant literature. This tool was structured into three parts:
**Part Ι: Patient's Sociodemographic and Health Relevant Data** The first section collected the patients' sociodemographic and clinical data, including age, gender, occupation, smoking status and key health‐related information, such as the date and reason for intensive care unit (ICU) admission, medical diagnosis, past medical history, ICU length of stay and the Modified Glasgow Coma Score (MGCS) score.
**Part ΙΙ: Ventilator Modalities Data** The second section focused on mechanical ventilation parameters, documenting details, such as the initiation date of mechanical ventilation, the type of artificial airway used, endotracheal tube size, ventilation mode and duration of mechanical ventilation.
**Part ΙΙΙ: Endotracheal Suctioning Data** The third section captured data related to endotracheal suctioning, including the size of the suction catheter, the type of catheter connector and the total duration of the suctioning procedure. The content validity of this tool was assessed by five experts in ICU nursing and medicine.
Tool ΙΙ: VAP Diagnostic Criteria Sheet
The second data collection tool utilised in this study was the ventilator‐associated pneumonia (VAP) diagnostic criteria sheet, adopted from Singh et al. [23], which was used to assess patients for clinical VAP diagnosis. This tool incorporates the Modified Clinical Pulmonary Infection Score (MCPIS), which is based on five clinical assessment parameters. Each parameter is assigned a score ranging from 0 to 2 and includes body temperature, white blood cell count, the purulence and quantity of tracheal secretions (categorised as rare, abundant or purulent abundant), oxygenation status (calculated as the ratio of arterial oxygen partial pressure [PaO2] to the fraction of inspired oxygen [FiO2]) and findings from chest radiography (classified as no infiltrates, diffuse infiltrates or localised infiltrates). The total MCPIS score is derived by summing the individual scores for each variable, resulting in a range from 0 to 10 for data analysis. This tool has been widely used in numerous studies to assess the clinical diagnosis of VAP indicating its validity and applicability. VAP was checked on Day 3 (early VAP) and again on Day 6 (late VAP). If a patient left the study after Day 3, they were not replaced, and only their Day 3 (E‐VAP) data were included in the analysis. Patients who stayed until Day 6 were also checked for late VAP (L‐VAP). As this was a feasibility study, the focus was not on how often VAP occurred, but on whether the intervention could be carried out as planned.

### 3.9 Outcome Measures

The outcomes of the study focused on the feasibility of the intervention, the feasibility of conducting the trial and patient outcomes (Box 2).

**BOX 2:** The outcomes of the study.

Feasibility of the intervention Evaluating the feasibility of using chlorhexidine as a flushing solution of endotracheal suction circuits in intensive care unit (ICU). Exploring the feasibility of using the suction catheter a single time for suctioning procedure. Exploring any potential adverse effects of the interventions. Feasibility of conducting the trial d Evaluating flexibility with resources utilisation (suction catheters, saline and chlorhexidine), for the conduction trail, and the number of eligible, recruited and withdrawn patients. e Identifying confounding factors which might affect the proposed trial (i.e., age, severity of disease and underlying diseases). Patients' outcomes f Ventilator‐associated pneumonia. g Length of ICU stays and mortality. Assessing different potential primary and secondary outcomes of the future trial h Investigating the impact of the proposed interventions on health economics.

### 3.10 Analysis

As a feasibility study, the statistical analysis adopted a descriptive approach. The primary objective of the analysis was to evaluate the feasibility of the intervention, determine the viability of conducting a full‐scale definitive trial, and summarise potential primary and secondary outcome measures.

To identify secondary patient outcomes, the collected quantitative data were coded, processed and statistically analysed using the IBM SPSS Statistics for Windows, version 24 (IBM Corp., Armonk, NY, USA). The results are presented as frequencies and percentages for categorical variables and as means with standard deviations for continuous variables.

## 4 Results

### 4.1 Recruitment and Retention

**FIGURE 1:**
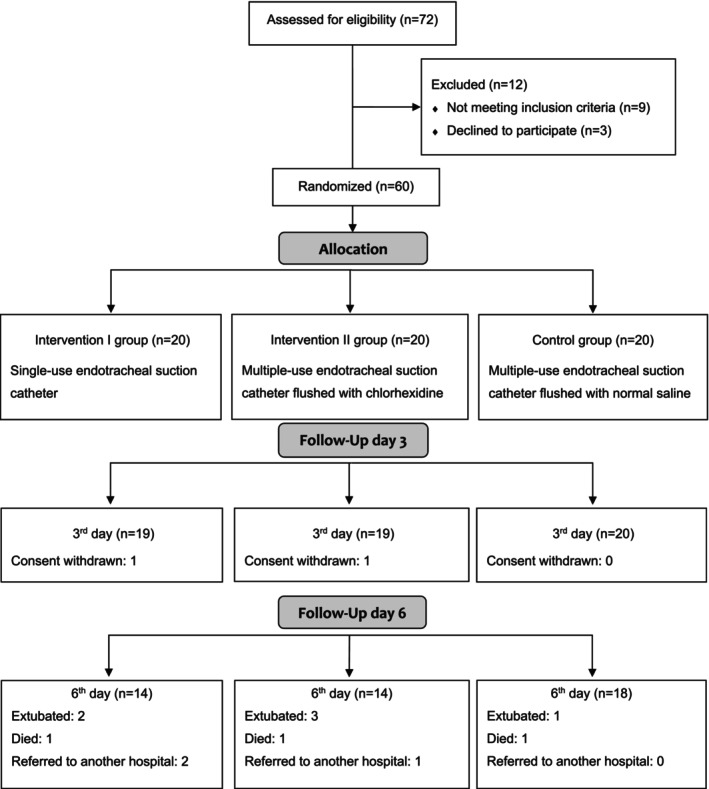
Flow diagram of study participants. Patients assessed for early VAP on Day 3; those who remained were also assessed for late VAP on Day 6. Patients who dropped out after Day 3 were not replaced, as the focus was on feasibility, not disease incidence.

A total of 60 participants were initially enrolled, but two patients were withdrawn from the study by their next of kin—one from the Intervention 1 group and one from the Intervention 2 group—resulting in a final sample of 58 participants. The CONSORT flow diagram (Figure 1) provides a summary of the recruitment process. The sealed envelope method for randomisation was successful and practical in the feasibility RCT. It was simple to use and helped keep the group allocation fair and hidden; therefore, it should be a good choice for the future RCT.

Retention was high across all groups, with 96.7% (*n* = 58) of participants completing the 3‐day follow‐up and 93.3% (*n* = 56) completing the 6‐day follow‐up. Retention rates were consistent across groups, with the Intervention 1 group (95% at 3‐day follow‐up, 70% at 6‐day follow‐up), Intervention 2 group (95% at 3‐day follow‐up, 70% at 6‐day follow‐up) and Control group (100% at 3‐day follow‐up, 90.0% at 6‐day follow‐up). All cases of non‐completion (*n* = 2) resulted from withdrawals initiated by the next of kin. The demographic characteristics of participants are detailed in Table 1.

**TABLE 1:** Sociodemographic and health relevant data of the study groups.

Variable	Intervention I	Intervention II	Control
	*n* (%)	*n* (%)	*n* (%)
Age (years)			
Mean ± SD	47.53 ± 14.30	50.37 ± 20.31	48.35 ± 15.45
Gender			
Males	11 (57.9%)	15 (78.9%)	13 (65.0%)
Females	8 (42.1%)	4 (21.1%)	7 (35%)
Smoking habit			
Current smoker	3 (15.8%)	5 (26.3%)	5 (25%)
Past smoker	3 (15.8%)	4 (21.1%)	3 (15%)
Non‐smoker	13 (68.4%)	10 (52.6%)	12 (60%)
Reasons for admission			
Respiratory	0 (0%)	0 (0%)	1 (5%)
Multiple injuries (Trauma)	10 (52.6%)	9 (47.4%)	5 (25%)
Cardiac	1 (5.3%)	0 (0%)	2 (10%)
Neurological	8 (42.1%)	10 (52.6%)	11 (55%)
Others (Toxicology)	0 (0%)	0 (0%)	1 (5%)
Past medical history			
Yes	10 (52.6%)	12 (63.2%)	12 (60%)
No	9 (47.4%)	7 (36.8%)	8 (40%)
Diabetes mellitus	9 (31.03%)	8 (25%)	10 (30.3%)
Hypertension	9 (31.03%)	11 (34.38%)	11 (33.34%)
Ischemic heart disease	6 (20.69%)	7 (21.88%)	4 (12.1%)
Renal failure	3 (10.34%)	3 (9.38%)	3 (9.09%)
Hepatic impairment	2 (6.89%)	3 (9.38%)	5 (15.2%)

*Note:* Data are presented as numbers (*n*), frequency (%) and Mean ± standard deviation (SD).

### 4.2 Adherence to and Refinement of the Intervention

Adherence to the intervention following the provided nurses' training was excellent, with 100% of participants receiving the designated intervention. The clinical lead nurse and PI observed nursing practices during the daytime, whereas the charge nurse conducted observations during night shifts to ensure adherence. The coloured labels applied to each group's participant files played a crucial role in ensuring continuity of data and preventing errors in delivering the correct intervention.

For participants in the Intervention 1 group, when the single‐use catheter was used, normal saline was administered when necessary to flush the circuit and prevent secretion build‐up from blocking the endotracheal tube. The ventilator modalities and endotracheal suction data of the study participants are presented in Table 2.

**TABLE 2:** Ventilator modalities and endotracheal suctioning data of the study groups.

Variable	Intervention I	Intervention II	Control
	*n* (%)	*n* (%)	*n* (%)
Artificial airway used			
Endotracheal tube	19 (100%)	19 (100%)	20 (100%)
Tracheostomy tube	0 (0%)	0 (0%)	0 (0%)
Intubation process			
Urgent	19 (100%)	19 (100%)	20 (100%)
Elective	0 (0%)	0 (0%)	0 (0%)
ETT size (mm)			
6.0–6.5	1 (5.3%)	0 (0%)	1 (5%)
7.0–7.5	11 (57.9%)	14 (73.7%)	11 (55%)
8.0–8.5	7 (36.8%)	5 (26.3%)	8 (40%)
Mode of ventilation			
Controlled	2 (10.5%)	1 (5.3%)	4 (20%)
Assisted	16 (84.2%)	15 (78.9%)	16 (80%)
Spontaneous	1 (5.3%)	3 (15.8%)	0 (0%)
Suction catheter size (Fr)			
12 fr	4 (21.1%)	4 (21.1%)	6 (30%)
14 fr	9 (47.4%)	12 (63.2%)	12 (60%)
16 fr	6 (31.5%)	3 (15.7%)	2 (10%)
Type of SC connector			
Standard connector	7 (36.8%)	6 (31.6%)	11 (55%)
Thumb control connector	6 (31.6%)	11 (57.9%)	7 (35%)
Fingertip control connector	6 (31.6%)	2 (10.5%)	2 (10%)
Duration of total suction time			
< 30 s	2 (10.5%)	1 (5.3%)	2 (10.5%)
30 s – 1 min	10 (52.6%)	12 (63.1%)	9 (47.4%)
> 1 min	7 (36.9%)	6 (31.6%)	8 (42.1%)
Frequency of using suction catheter			
Single time	19 (100%)	0 (0%)	0 (0%)
Multiple times	0 (0%)	19 (100%)	20 (100%)
Flushing solution used			
Normal saline	19 (100%)	0 (0%)	20 (100%)
Chlorhexidine	0 (0%)	19 (100%)	0 (0%)

*Note:* Data are presented as numbers (*n*) and frequency (%).

Abbreviations: ETT; endotracheal tube; Fr, French gauge; MV, mechanical ventilation; mm, Millimetre; SC, suction catheter.

### 4.3 Feasibility of Inclusion/Exclusion Criteria

During the feasibility phase, we observed that applying the exclusion criterion related to prior endotracheal suctioning was challenging. Although the study aimed to exclude patients who had already undergone multiple suctioning procedures before enrolment, in practice, some patients received at least one suctioning event as part of routine ICU care before deferred consent could be obtained. This highlighted the difficulty of implementing this criterion within the first 24 h of admission, especially in a critical care setting where early intervention is standard. As such, this exclusion criterion may need to be reconsidered or adapted in future studies to better align with the timing of patient enrolment and standard clinical workflows.

### 4.4 Feasibility of Outcome Assessment

The primary outcome measures included parameters, such as the feasibility of using chlorhexidine as a flushing solution, the feasibility of using single‐use suction catheters, and resource availability, as summarised in Box 3. The secondary outcome measure includes the patients' outcomes and focused on length of ICU stay, average Glasgow Coma Scale (GCS), average MCPIS and the incidence of VAP, which are summarised in Table 3.

**BOX 3:** Comparison of intended measures and findings.

Parameter	Intended measurement	Findings
Feasibility of chlorhexidine flushing	Determine whether chlorhexidine can be used as a flushing solution for suctioning	Feasible; nurses used it successfully and found it a useful way of using chlorhexidine rather than oral care
Feasibility of single‐use suction catheters	Evaluate whether using a suction catheter only once per procedure is practical	Feasible; nurses and hospital administrative accepted the practice as long as there was sufficient supply
Resource availability	Assess the availability of suction catheters, saline and chlorhexidine	Resource limitations posed a constraint; therefore, additional saline, chlorhexidine and suction catheters were purchased to facilitate the study conduction and ensure adherence to the interventions
Patient recruitment and retention	Evaluate number of enrolled, withdrawn and retained participants	60 patients enrolled: 2 withdrawn by next of kin, leaving 58 participants
Influence of confounding factors	Assess whether age, disease severity or underlying conditions affected results	There were no apparent differences between study groups, suggesting that these factors were unlikely to have influenced the results
Mortality	To assess the impact of the proposed interventions on patients' mortality	No significant differences were observed between the study groups, with 20 participants enrolled in each. Mortality rates remained similar across groups, with only one participant death per group, indicating that the intervention might not be likely to influence mortality.
Health economics	Investigating the impact of the proposed interventions on health economics.	No significant differences were observed between the two intervention groups in terms of funding expenditure; the costs of chlorhexidine, saline and suction catheters used in the study were comparable.

**TABLE 3:** Secondary outcomes findings of the study groups.

Variable	Intervention I	Intervention II	Control	Significance^a^
	*n* (%)	*n* (%)	*n* (%)	
Length of ICU stay (days)				
3–4 days	1 (5.3%)	2 (10.5%)	1 (5%)	95% CI 0.354–1.298
5–6 days	4 (21.1%)	3 (15.8%)	1 (5%)	
≥ 7 days	14 (73.6%)	14 (73.6%)	18 (90%)	
Average MGCS (Mean ± SD)				
Day 1	19 (6.95 ± 0.91)	19 (7.11 ± 1.24)	20 (7.0 ± 1.81)	95% CI 0.831–0.984
Day 2	19 (6.53 ± 1.02)	19 (6.79 ± 1.44)	20 (6.40 ± 1.51)	
Day 3	19 (6.42 ± 1.26)	19 (6.63 ± 1.83)	20 (6.10 ± 1.94)	
Day 4	19 (6.21 ± 1.69)	19 (6.16 ± 1.74)	20 (5.95 ± 2.16)	
Day 5	17 (5.94 ± 1.71)	15 (6.20 ± 1.69)	19 (5.95 ± 2.04)	
Day 6	14 (5.57 ± 1.99)	14 (6.43 ± 2.14)	18 (5.61 ± 2.17)	
MCPIS (Mean ± SD)				
Day 1	19 (1.53 ± 1.50)	19 (1.42 ± 0.96)	20 (1.55 ± 1.28)	95% CI 0.612–0.874
Day 3	19 (4.73 ± 1.82)	19 (3.74 ± 1.73)	20 (4.70 ± 1.89)	
Day 6	14 (5.29 ± 2.52)	14 (4.86 ± 1.83)	18 (6.05 ± 2.63)	
Incidence of VAP				
Day 3 early VAP				
VAP	2 (25%)	2 (20%)	3 (37.5%)	RR intervention I vs Control = 0.67 (95% CI: 0.12–3.61), RR intervention II vs Control = 0.53 (95% CI: 0.10–2.79)
Not VAP	6 (75%)	8 (80%)	5 (62.5%)	
Day 6 late VAP				
VAP	4 (36.4%)	3 (33.3%)	5 (41.7%)	RR intervention I vs Control = 0.87 (95% CI: 0.29–2.60), RR intervention II vs Control= 0.80 (95% CI: 0.22–2.87)
Not VAP	7 (63.6%)	6 (66.6%)	7 (58.3%)	
Total VAP				
VAP	6 (31.6%)	5 (26.3%)	8 (40%)	RR intervention I vs Control = 0.79 (95% CI: 0.51–1.23), RR intervention II vs Control = 0.66 (95% CI: 0.42–1.03)
Not VAP	13 (68.4%)	14 (73.7%)	12 (60%)	

*Note:* Data are presented as numbers (*n*) and frequency (%).

Abbreviations: MGCS, Modified Glasgow Coma Score, MCPIS, Modified Clinical Pulmonary Infection Score; VAP, ventilator‐associated pneumonia.

^a^ The 95% CI represents the relative risk (RR) of VAP for the intervention groups compared with control. All other variables (e.g., MGCS, MCPIS) retain their means ± SD with CIs representing mean differences. VAP checked on Day 3 for early VAP and Day 6 for late VAP.

### 4.5 Adverse Events

The safety of the intervention was evaluated by monitoring and comparing the occurrence of any adverse events across the three study groups. Throughout the study, no adverse events were observed or reported. Potential or expected adverse effects included chlorhexidine sensitivity, as well as adverse reactions related to the suctioning procedure or secretion removal, such as transient hypoxia, hemodynamic fluctuations, airway trauma, accidental extubation or bronchospasm.

### 4.6 Sample Size Estimation for Future RCT

Based on recruitment data from this feasibility RCT, it appears feasible to enrol between 200 and 400 participants in a future trial within this population. To determine the appropriate sample size, we conducted a statistical power analysis to detect a significant difference in the incidence of VAP, using a two‐sided test with a significance level (α) of 0.05 and statistical power (1–β) of 0.95. This sample size range should ensure sufficient statistical power to detect clinically meaningful differences.

The expected effect size was derived from relevant published studies on VAP prevention strategies using suction circuit flushing [2], as well as from preliminary data obtained in this feasibility RCT. The effect size was estimated using proportions (Cohen's *h*) or risk difference formulas for binary outcomes.

## 5 Discussion

The aim of this study was to determine the feasibility of investigating the effect of single‐use or multiple‐use open endotracheal suction catheters flushed with chlorhexidine compared to multiple‐use open endotracheal suction catheters flushed with normal saline on VAP incidence in mechanically ventilated patients to determine methods for the design of a conclusive RCT.

Our feasibility RCT met the established feasibility criteria and demonstrated that single‐use versus multiple‐use endotracheal suctioning catheters flushed with chlorhexidine interventions can be successfully implemented in ICUs located in resource‐limited countries once provided there is enough supply. The feasibility parameters provided valuable insights to enhance the design of a future definitive trial.

Recruitment to the study proceeded efficiently and without significant challenges. However, two patients were withdrawn from the study after initial enrolment, despite informed consent being obtained and their relatives having 24 h to decide on participation. The withdrawal was initiated by a different family member who had not reviewed the consent form, even though a copy was attached to the patient's file. Nevertheless, this decision was respected, and the patients were withdrawn from the study in accordance with the World Medical Association Declaration of Helsinki [24]. This highlights the importance of making sure that all key relatives are properly informed and involved in the consent process, especially in studies involving critically ill patients, where family members often have an important role in making decisions [25, 26]. Future RCTs may benefit from measures, such as direct communication with multiple family members or providing additional time for deliberation to address such ethical complexities.

After receiving training, nurses became familiar with the study interventions and the correct procedures for implementing them. However, when performing the interventions at the bedside, they faced challenges in identifying which patients belonged to which study group. To address this issue, coloured labels were added to patients' files to ensure that the correct intervention was delivered to the appropriate patient. Initially, labels were placed on patient beds, but this approach was not approved by the infection control team as it does not match their adopted protocols [27]. As a result, the labels were instead placed in patient files, a solution that was accepted by both the infection control team and hospital management.

The implementation of the intervention was influenced by the resource‐limited ICU context, including supply availability, staffing constraints and institutional protocols. These factors affected both logistics and adherence, suggesting that although the intervention is feasible in similar settings, the results may not be directly generalisable to high‐resource environments. Future studies should consider context‐specific adaptations and explore variability across different healthcare settings.

A study by Eid et al. [2] conducted in Egypt investigated a similar topic and found that flushing the suction circuit with chlorhexidine can reduce the incidence of VAP. However, their research focused on the repeated use of suction catheters with chlorhexidine flushing only and did not consider the impact of single‐use catheters, which align with international guidelines. Our study considered this point and demonstrated that the cost of single‐use catheters is comparable to that of reusing suction catheters with chlorhexidine flushing, suggesting that these two interventions may have similar economic impacts and could be considered equally cost‐effective.

The study protocol [18] did not specify whether a flushing solution should be used for the group utilising single‐use suction catheters. As a result, nurses encountered situations where secretions clogged the suction tubing, necessitating the use of a flushing solution. In these cases, saline was used, as it is considered standard care. Future RCTs should further investigate the necessity and effectiveness of flushing solutions in this context.

Regarding the secondary outcome measures, particularly the incidence of VAP, the findings in this study show a VAP incidence of 31.6%, 26.3% and 40% in Intervention I group, Intervention II group and the Control group, respectively. Although the results indicate potential differences in VAP incidence between groups, statistical analysis was not measured, as the primary aim of this feasibility RCT was to assess the practicality and feasibility of the intervention rather than to assess disease incidence.

## 6 Limitations

This study has several limitations that should be considered when designing a full‐scale RCT. Firstly, the study was conducted in a single hospital, which limits the generalisability of the findings. To enhance external validity, a multicentre RCT would be necessary to ensure the results are applicable across diverse settings. Another limitation is the issue of blinding, which was not feasible in this study. However, in a definitive RCT, blinding could be implemented by using identical containers for both the Intervention II and Control groups, with different solutions, to minimise bias and outcome assessment could and should have been performed by a person blinded to patient allocation. The data collection should be carried out by an assigned staff nurse or the clinical lead, as having the PI collect the data could introduce bias. Another limitation is that initial verbal consent had to be accepted in order to commence data collection during the first endotracheal suction attempt, due to the urgency of the clinical context. Additionally, due to resource constraints, the incidence of VAP was assessed using the MCPIS, whereas bronchoalveolar lavage (BAL) with a bacterial count threshold of > 10^4^ colony‐forming units per millilitre (CFU/ml) remains the most accurate diagnostic method. Finally, the study did not include laboratory testing to analyse bacterial species in the suction collection jar following chlorhexidine flushing, which could have provided insights into its impact on microbial flora within the circuit. Finally, an important potential source of bias that should be acknowledged is the suction catheter size (French gauge) and the type of suction catheter connector used. These variables could influence the effectiveness of suctioning and flushing and thus affect the outcomes. Future studies should standardise or account for these factors to minimise bias.

## 7 Conclusion

This study has assessed key feasibility parameters, demonstrating that a future RCT comparing single‐use and multiple‐use open endotracheal suction catheters with a chlorhexidine‐based flushing intervention versus standard care of multiple‐use open endotracheal suction catheters with a chlorhexidine‐based flushing is viable in resource‐limited ICU settings. Although this feasibility trial was not designed to evaluate the efficacy of the intervention, it successfully tested methodologies to identify existing limitations in study design and the literature, refining the approach for a future definitive trial. The findings suggest potential improvements in nursing practices within resource‐constrained environments. To build on this, a full‐scale RCT is recommended to assess the clinical‐ and cost‐effectiveness of the intervention, with a particular focus on the impact of this intervention on mechanically ventilated patients' outcomes. This would generate robust evidence to guide evidence‐based practices and inform policy in ICU settings with limited resources.

## Author Contributions

M.H.E. and J.M.L. contributed to the design of the study. M.H.E. performed the data collection and conducted the analysis. K.H., P.S. and J.M.L. contributed to the interpretation and synthesis of the data. The initial draft of the manuscript was prepared by M.H.E. K.H., P.S. and J.M.L. provided critical feedback and revisions to the manuscript. All authors reviewed the final version of the manuscript and approved it for submission.

## Ethics Statement

This study has been reviewed and approved by the following ethics committees: the Faculty of Health Research Ethics and Integrity Committee at the University of Plymouth, United Kingdom (Reference: 4333) and the Research Ethics Committee at the Faculty of Nursing, Mansoura University, Egypt (Reference: 411/2023).

## Consent

The research ethics committees have reviewed and approved the informed consent forms and participant information sheets of this study.

## Conflicts of Interest

The authors declare no conflicts of interest.

## Supporting information


**Data S1:** Nurses training package.

**Data S2:** Data collection tools.

## Data Availability

The data that support the findings of this study are available from the corresponding author upon reasonable request.
