# Supplementary material for: Single‐Use Versus Multiple‐Use Endotracheal Suction Catheters in Mechanically Ventilated Patients: A Feasibility Randomised Controlled Trial
Source: Nurs Crit Care. 2026 Jan 7;31(1):e70237. doi: 10.1111/nicc.70237 (PMC12779588; doi:10.1111/nicc.70237)
Supplement: Supplementary file 2 — Data S2: Data collection tools. [file NICC-31-0-s001.docx]

**Single-use versus Multiple-use Endotracheal Suction Catheters in Mechanically Ventilated Patients: A Feasibility Randomised Controlled Trial**

##

## Supplementary Material II: Data Collection Tools

- **Patient's Name:**

**Patient's Code:**

- **Admission Number:**
- **Study Group:**

| **Intervention I** |
| --- |
| **Intervention II** |
| **Control** |

**Tool I: Mechanically Ventilated Patient Assessment Tool**

**Part 1: Patient's Socio-demographic and Health Relevant Data**

1. **Patient's Characteristics:**

| 1. **Age:**   18-20  31-40  51-60 | | |  | 21- 30  41-50  Above 60 |  |
| --- | --- | --- | --- | --- | --- |
| 1. **Gender** | | |  |  |  |
| Male | | |  |  |  |
| Female | | |  |  |  |
| Other | | | Specify… | |  |
| 1. **Occupation:** | | |  |  |  |
| Employee | | |  | Farmer/Worker |  |
| Housewife | | |  | Retired |  |
| Other (specify): | | |  |  |  |
| 1. **Smoking Habits:** | | |  |  |  |
| Current Smoker |  | Past Smoker |  | Non - Smoker |  |

1. **Health Relevant Data:**

| 1. **Date of admission to ICU: / /** | |
| --- | --- |
| 1. **Reason for admission:** | |
| Respiratory Failure | Multiple Injury |
| Cardiac Disease | Renal Disease |
| Neurological Disease | Others Specify……………… |
| 1. **Medical diagnosis:** |  |
| 1. **Past medical history:** |  |
| Diabetes Mellitus | Hypertension |
| Ischemic heart disease | Renal failure |
| Hepatic impairment | Others specify……………… |

1. **Duration of ICU Stay**

| 1-2 days | 3-4 days |
| --- | --- |
| 5-6 days | ≥7 days |

1. **Level of Consciousness Based on Modified Glasgow Coma Scale (MGCS):**
2. **On admission:**
3. **Daily assessment:**

| Day/shift | 1^st^ day | 2^nd^ day | 3^rd^ day | 4^th^ day | 5^th^ day | 6^th^ day |
| --- | --- | --- | --- | --- | --- | --- |
| MGCS |  |  |  |  |  |  |

**Part II: Ventilator Modalities Data**

1. **Mechanical Ventilation Initiation Date:**
2. **Artificial airway**

| Endotracheal Tube | Tracheostomy |
| --- | --- |
| Laryngeal Mask | Others |

1. **Intubation Process**

| Urgent | Elective |
| --- | --- |

1. **Size of Endotracheal Tube**

| 5-5.5 | 6-6.5 |
| --- | --- |
| 7-7.5 | 8-8.5 |

1. **Mode of Ventilation**

| Controlled |
| --- |
| Assisted |
| Spontaneous |

1. **Duration of Mechanical Ventilation**

| 1-2 days | 3-4 days |
| --- | --- |
| 5-6 days | ≥7 days |

**Part III: Endotracheal Suctioning Data**

| 1. **Size of Suction Catheter** |  |
| --- | --- |
| ≤10 Fr | 12 Fr |
| 14 Fr | 16 Fr |
| 18 Fr | 20 Fr |
| 1. **Type of Suction Catheter Connector** | |
| Standard Connector |  |
| Thumb Control Connector |  |
| Fingertip Control Connector |  |
| 1. **Duration of Total Suction Time** |  |
| ˂ 30 Seconds |  |
| 30 Seconds -1 Minute |  |
| ˃ 1 Minute |  |
| 1. **Frequency of Using Suction Catheter** |  |
| Single-used | Multiple Used |
| 1. **Flushing Solution Used** |  |
| Normal Saline | Chlorhexidine |

**Tool ΙΙ: VAP Diagnostic Criteria Sheet**

**The Modified Clinical Pulmonary Infection Score**

**(Modified CPIS)**

| **CPIS Elements** | **Range** | **Score** | **Day 1** | **Day 3** | **Day 6** |
| --- | --- | --- | --- | --- | --- |
| **Temperature** | ≥ 36.5 and ≤ 38.4 | 0 |  |  |  |
|  | ≥ 38.5 and ≤ 38.9 | 1 |  |  |  |
|  | ≥ 39 and ≤ 36 | 2 |  |  |  |
| **Blood Leukocytes Count, per mm^3^** | 4,000 – 11,000 | 0 |  |  |  |
|  | ˂4,000 or ˃11,000 | 1 |  |  |  |
|  | ˂4,000 – ˃11,000 + band forms ≥ 500 | 2 |  |  |  |
| **Tracheal Secretions** | Rare | 0 |  |  |  |
|  | Abundant | 1 |  |  |  |
|  | Abundant + Purulent | 2 |  |  |  |
| **Oxygenation PaO_2_/FiO_2_** | ˃ 240 or ARDS | 0 |  |  |  |
|  | ≤ 240 and no evidence of ARDS | 2 |  |  |  |
| **Chest X-ray Infiltrates** | No infiltrates | 0 |  |  |  |
|  | Diffused | 1 |  |  |  |
|  | localized | 2 |  |  |  |
| **Total Score** | **˃ 5 = VAP** |  |  |  |  |
